# Ambient air pollution exposure and emergency department visits for substance abuse

**DOI:** 10.1371/journal.pone.0199826

**Published:** 2018-06-29

**Authors:** Mieczysław Szyszkowicz, Errol M. Thomson, Ian Colman, Brian H. Rowe

**Affiliations:** 1 Population Studies Division, Health Canada, Ottawa, Canada; 2 Hazard Identification Division, Health Canada, Ottawa, Canada; 3 School of Epidemiology, Public Health and Preventive Medicine, University of Ottawa, Ottawa, Canada; 4 Department of Emergency Medicine, Faculty of Medicine & Dentistry, University of Alberta, Edmonton, Canada; 5 School of Public Health, University of Alberta, Edmonton, Canada; Telethon Institute for Child Health Research, AUSTRALIA

## Abstract

There is growing evidence supporting the notion that exposure to air pollution can contribute to cognitive and psychiatric disorders, including depression and suicide. Given the relationship between exposure to acute stressors and substance abuse, the present study assessed the association between exposure to ambient air pollution and emergency department (ED) visits for alcohol and drug abuse. ED visit data selected according to International Classification of Disease (ICD-9) coding 303 (alcohol dependence syndromes) and 305 (non-dependent abuse of drugs) were collected in five hospitals in Edmonton, Canada. A time-stratified case crossover design was used. Conditional logistic regression was applied to calculate odds ratios (OR) and 95% confidence intervals (95% CI). Season, temperature, and relative humidity were adjusted for using natural splines.

Results are reported for an increase in pollutant concentrations equivalent to one interquartile range (IQR). Statistically significant positive associations with substance abuse were observed for CO, NO_2_ and particulate matter with an aerodynamic diameter less than 10 μm (PM_10_) and 2.5 μm (PM_2.5_). The strongest results were obtained in the cold period (October–March) for 1-day lagged CO (OR = 1.03, 95% CI: 1.01, 1.05, IQR = 0.4 ppm) and NO_2_ (OR = 1.04, 95% CI: 1.01, 1.07, IQR = 12.8 ppb); ORs were also significant for CO and NO_2_ with lags of 2 to 6 days and 2 to 7 days, respectively. The study suggests that, even at low levels, increases in ambient CO, NO_2_, and PMs are associated with increased hospital admissions for substance abuse, possibly as a result of impacts of air quality on mental health or depression.

## Introduction

Despite considerable interest in the effects of air pollution on human health, research on the impact of ambient air pollution on mental illness is still in its infancy. The literature that does exist suggests associations between air pollution and a variety of disordered behaviors, including depression and suicide [1–6]. Supporting these findings, a recent animal study [7] indicated that after 10 months of exposure to air pollution at levels similar to those faced by many people daily, mice showed signs of depression, anxiety, and learning difficulties. In another study, exposure of mice to concentrated ambient particles increased their propensity to seek immediate reward [8]. Air pollutant exposure can acutely impact systems implicated in neurobehavioral disorders, as evidenced by rapid activation of the hypothalamic-pituitary-adrenal stress axis and downstream effects upon exposure to pollutants [9–11]. Together with a growing number of studies showing that the brain is a potential target of adverse effects of pollutant exposure [12], these findings support the hypothesis that ambient air pollutants can impact behavior.

There is an established link between substance abuse and mental disorders such as depression. Emergency department (ED) presentations for substance abuse may be manifestations of mental health issues such as depression [13,14]. If air pollutants do indeed affect the central nervous system and provoke psychiatric symptoms [15], one might expect a corresponding increasing in hospital presentations and admissions for drug and alcohol abuse.

In this study, we use a case-crossover design [16] to investigate the relationship between exposure to air pollutants and the number of ED visits for substance abuse. As a second method to study potential non-linear relations, we used statistical methodology to fit a parametric curve for the concentration-response model [17]. Such a curve represents concentration-response risks along the range of exposure levels and it is determined as the best approximation in the considered class of monotonic functions. The results presented in this study show potential triggering effects of air pollution on hospital admissions for substance abuse.

## Materials and methods

### Study subjects

The data on ED visits were provided by Alberta Health Services–Edmonton Zone for all five major acute care hospitals in the Edmonton area. Edmonton has an administratively-linked and academically-focused health care system and is part of one of the largest integrated health regions in Canada. The system provides complete health services to approximately one million residents. The study sample included patients served by 5 hospitals in Edmonton between April 1, 1992 and March 31, 2002. In this 10 year time period, 2,951,878 diagnosed ED visits were recorded.

### Substance use disorders

The primary outcome in this study was a recorded diagnosis of a substance use disorder during an ED visit. During the study all ED charts were coded by trained medical record nosologists using *The International Classification of Diseases*, *9th Revision* (ICD-9) codes. As a main measurement of health outcomes, we used substance use disorder identified by two ICD-9 codes. Visits that received a diagnosis of alcohol dependence syndromes (ICD-9: 303) or nondependent abuse of drugs (ICD-9: 305) were considered cases of substance use disorders. ED visits for dependent abuse of drug (ICD: 304) were not considered, as such visits were assumed to be mainly triggered and driven by addiction.

### Air pollution and meteorological data

The environmental data were measured, recorded and provided by Environment Canada. Ambient air pollutants were measured by the following techniques: carbon monoxide (CO)—nondispersive infrared spectrometry, nitrogen dioxide (NO_2_)—chemiluminescence, ozone (O_3_)—chemiluminescence/ultraviolet photometry, and sulphur dioxide (SO_2_)—coulometry/ultraviolet fluorescence. Ambient particulate matter (PM) with median diameter ≤2.5 and ≤10 μm (PM_2.5_ and PM_10_, respectively) concentrations were measured using tapered element oscillating microbalance instruments (See NAPS Web site: https://www.ec.gc.ca/rnspa-naps/).

In the period of the study four ambient air pollutants CO, NO_2_, O_3_, and PM_2.5_ were measured by three stations (northwest (N), central (C), and east (E)), PM_10_ by two stations (C and N), and SO_2_ only by one station (E). The largest distance between monitors is 11 km. The concentrations for ambient PM_10_ were measured for the period: January–December 1994 and March 1995-March 2002; and the concentrations for ambient PM_2.5_, for the period from April 1998 to March 2002. When data were available from two or three monitoring stations, they were averaged. For each monitoring station, the daily mean concentration was represented as the average of 24 hourly measurements. If more than 25% of daily (24 hourly values) measurements were missing, then for this monitor the whole day was assumed missing. Environment Canada also provided hourly data per day for relative humidity and temperature for the city of Edmonton, Alberta, Canada. We estimated the daily levels for the weather parameters temperature (dry bulb) and relative humidity by averaging hourly data over 24-hour measurement periods.

### Statistical analyses: Case-crossover approach

The case-crossover (CC) study design is an epidemiological approach [16] in which the case serves as his/her own control. It is an expansion of the case-control method, which is based on comparing exposures of the same subject in various time periods. This statistical method is mainly applied to study rare acute health events likely connected with short-term transient factors [18]. The CC method allows for the generation of unbiased conditional logistic regression estimates [19]. It is also well matched for studying lagged effects when short time intervals separate a change in exposure from the potential change in the odds of the considered health event. Finally, the CC method allows for persons to serve as their own controls. As a result, this technique compensates by design for locally time-invariant influences that may confound the relationship between air pollution and substance use disorders, such as sex, age, social position or chronic physical health conditions (comorbidity). Following established practice, we selected for each case day all the other day-of-week matched days in the same month that included the case day. This statistical approach results in 3 or 4 controls periods for each case [19]. In this way slowly changing confounding factors (such as patients’ health condition, socio-economic position, long-term trends, and seasonal effects) should not vary drastically between the case day and the control days. The generated estimates were reported as odds ratios (OR) with corresponding 95% confidence intervals (CI), which signify the ratios of odds of the considered health event for two levels of exposure differing by a conventional unit. The CC method was realized using the procedure PHREG in SAS, (SAS Institute Inc., SAS 9.1). Temperature and relative humidity in the constructed models were used in the form of natural splines of three degrees of freedom. Their values in the models were lagged by the same number of days as the air pollutant. As a standard, one interquartile range (IQR, from 75^th^ to 25^th^ percentile) in the exposure data represents the unit in change of the air pollution exposure. The associations were tested for exposure from lag 0 (same day exposure) to lag 7 (7 days before ED visit), with a p-value of 0.05 considered significant.

No patient-level identifying information was accessed as part of this study.

### Non-linear associations

In addition to the case-crossover approach, we performed a separate analysis to further interrogate the data. This study was mainly performed as a sort of sensitivity analysis and we focused on one air pollutant. To investigate potential non-linear associations (concentration curve-response) between exposure to carbon monoxide and odds ratios for ED visit data, we used a statistical technique that, in the case of positive associations (positive slope), estimates the parametric curve, which is monotonic and non-decreasing with concentration [17]. The case-crossover method and various concentration transformations are applied to realize this methodology [17]. Among the transformations considered, the best one (based on the estimated log-likelihood values) was chosen and finally used.

### Ethics

The Health Research Ethics Board of the University of Alberta approved this main study protocol. The study was conceived and designed after March 31, 2002 (i.e., it is a retrospective study examining data produced between April 1, 1992 and March 31, 2002). These data are used in the retrospective study related to ambient air pollution exposure and health outcomes.

## Results

**Fig 1:**
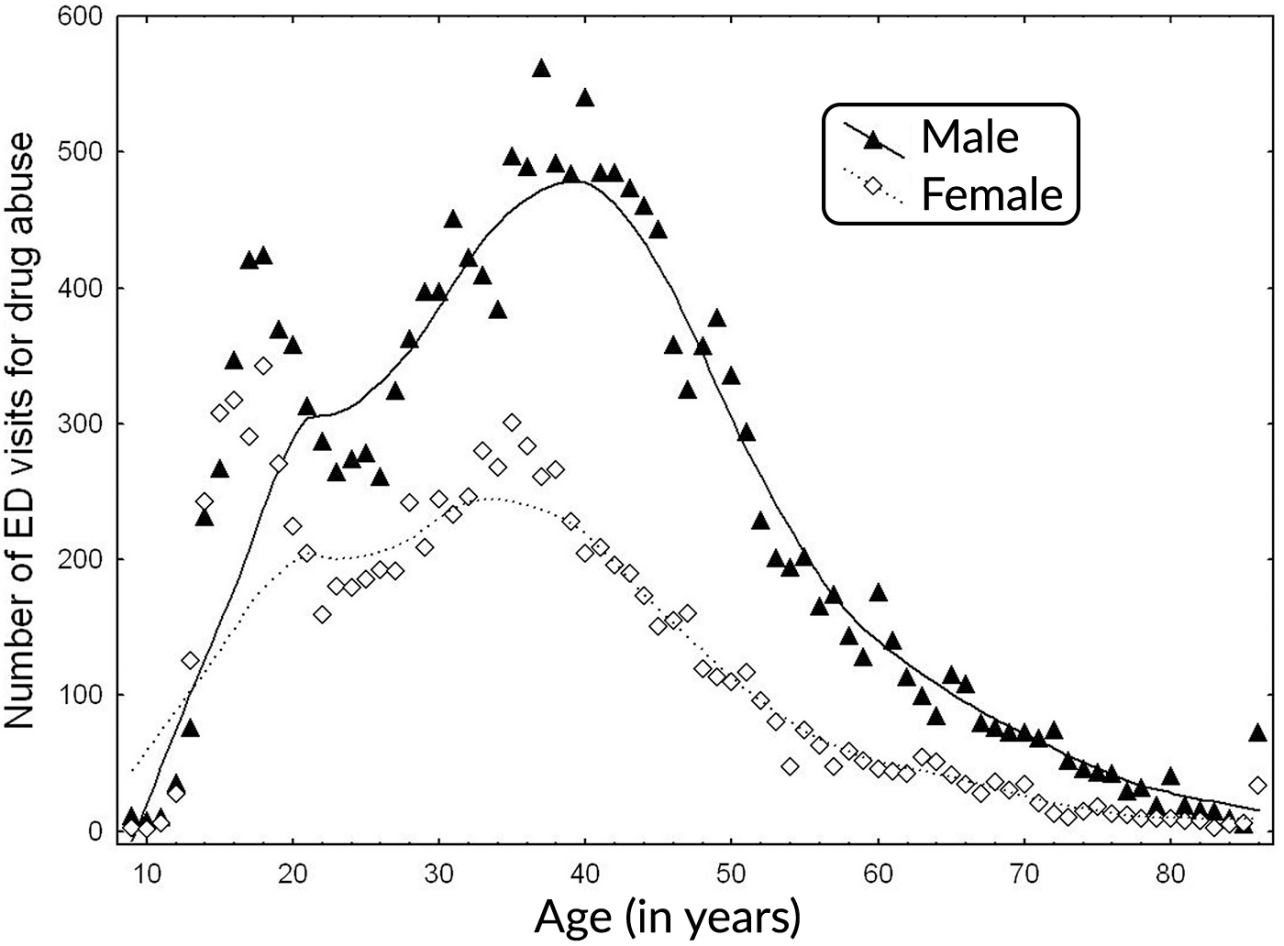
Number of ED visits for age group, at five Edmonton emergency departments by age and sex. Age is presented as age in years, however visits by those aged <10, or >85 were combined to single points (in 9 and 86). Filled triangles denote visits by males, empty diamonds visits by females. Edmonton, Canada, 1992–2002.

There were 27,534 diagnosed visits for substance abuse disorders over the 10-year study period; among them 9,547 (34.6%) cases were females and 17,987 (65.4%) cases were males (see Fig 1). Of these, 5,921 cases presented with alcohol dependence (4,207 cases were males), and 21,613 cases presented with non-dependent substance abuse (13,780 cases were males). Frequencies of ED visits by age and sex are shown on Fig 1. In general, the frequency of ED visits was higher for males than females across all age groups. The frequency of days with visits, expressed as the percentage of all days, was as follows: 1.4% of days with one visit, 4.1% with two, 6.5% with 3, 9.1% with 4, 10.5% with 5, 6, 7, and 8 visits, 9.4% with 9 visits, and 7.4% with 10 visits. The frequency of days with 16+ visits was below one percent. There were 20 days over a 10 year period of the study with no ED visits related to abuse of substances. The percentages of all visits per day of the week were 16.3 (Sunday), 10.9 (Monday), 12.2, 12.6, 13.0, 15.5, and 19.5% (Saturday), respectively. Starting from January, ED presentation by months ranged from a low of 7.2% (February) to a high of 9.2% (October).

Comparison of mean air pollutant concentrations across the different monitoring stations showed reasonably good correlations are presented in Table 1. The correlation coefficients between the daily mean values of air pollutant levels are presented in Table 2. All estimated coefficients were statistically significant (i.e., P-value < 0.05) with one exception for the correlation between ambient ozone and coarse particulate matter (O_3_ and PM_10_, with the coefficient = 0.03). Tables 3 and 4 show statistics of air pollutants, where Table 4 summarizes values by two periods; warm (April-September) and cold (October-March).

**Table 1:** The correlation between air pollution levels for three monitoring stations (E–east, N–northwest, C–central). Edmonton, Canada, 1992–2002.

Variable (unit)	Days	Station E-N	Station E-C	Station N-C
CO (ppm)	3,512	0.79	0.78	0.80
NO_2_ (ppb)	3,431	0.74	0.80	078
SO_2_ (ppb)	3,652	a	a	a
O_3_ (ppb)	3,552	0.85	0.89	0.88
PM_10_ (μg/m^3^)	835	b	b	0.81
PM_2.5_ (μg/m^3^)	506	0.79	0.87	0.88

a—only one station was used (E); b–values for 2,813 days are only from one station (C); values for PM_2.5_ for 1,442 days are from various stations.

**Table 2:** The estimated correlations calculated pairwise. Upper triangle represents the coefficients for all data; lower triangle for cold period data. Edmonton, Canada, 1992–2002.

Pollutant (unit)	CO	NO_2_	SO_2_	O_3_	PM_10_	PM_2.5_
CO (ppm)	1	0.78	0.43	-0.55	0.32	0.43
NO_2_ (ppb)	0.74	1	0.47	-0.53	0.35	0.39
SO_2_ (ppb)	0.34	0.46	1	-0.27	0.22	0.21
O_3_ (ppb)	-0.59	-0.52	-0.24	1	0.03	-0.07
PM_10_ (μg/m^3^)	0.54	0.48	0.27	-0.29	1	0.76
PM_2.5_ (μg/m^3^)	0.71	0.57	0.28	-0.43	0.68	1

**Table 3:** Number of days with data, mean values, standard deviation (SD), median, max–maximum recorded value, interquartile range (IQR = Q3-Q1, Q1 – 25^th^ percentile, Q3 – 75^th^ percentile). Edmonton, Canada, 1992–2002.

Variable (unit)	Days	Mean	SD	Median	Max	IQR	Q3
CO (ppm)	3,652	0.7	0.4	0.6	4.5	0.4	0.8
NO_2_ (ppb)	3,652	21.9	9.4	19.7	67.6	12.8	27.6
SO_2_ (ppb)	3,652	2.6	1.8	2.2	16.3	2.3	3.5
O_3_ (ppb)	3,652	18.6	9.3	17.8	50.7	14.0	25.2
PM_10_ (μg/m^3^)	2,813	22.6	13.1	19.4	137.4	15.0	28.3
PM_2.5_ (μg/m^3^)	1,444	8.5	6.2	7.2	103.1	6.2	10.9
Temperature (^o^C)	3,652	3.9	11.9	5.4	26.5	17.9	13.9
Relative humidity (%)	3,652	66.0	13.6	66.1	98.5	18.5	75.6

**Table 4:** Number of days with data (NW–left-hand part in warm season, NC–right-hand part in cold season), mean values, standard deviation (SD), median, Max–maximum recorded value. Edmonton, Canada, 1992–2002.

Pollutant (unit)	NW	Mean	SD	Max	NC	Mean	SD	Max
CO (ppm)	1830	0.6	0.2	3.2	1822	0.9	0.5	4.8
NO_2_ (ppb)	1830	16.5	5.6	43.0	1822	27.2	9.3	67.5
SO_2_ (ppb)	1804	2.1	1.6	13.6	1812	3.1	2.0	16.6
O_3_ (ppb)	1830	23.4	8.3	50.9	1822	13.9	7.7	49.8
PM_10_ (μg/m^3^)	1441	23.9	13.8	137.0	1372	21.0	12.1	103.2
PM_2.5_ (μg/m^3^)	711	8.7	7.0	102.4	729	8.3	5.2	45.4

**Fig 2:**
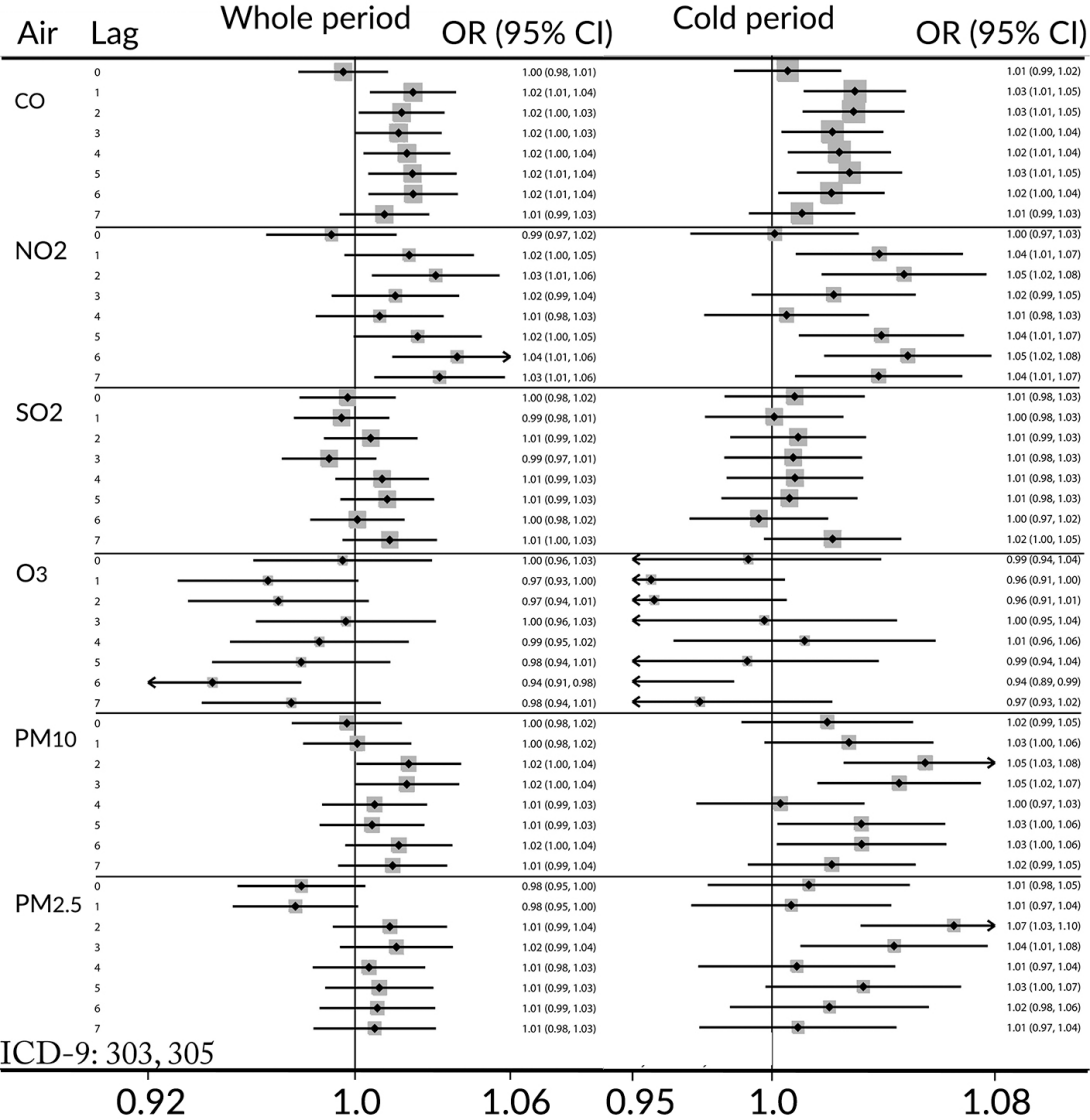
ORs and 95% CIs for ED visits for substance use disorders for an IQR change in the levels of the named pollutant. The ORs are reported for same day (0) as well as lags 1–7 days. Left-hand panel—whole period: January—December, right-hand panel—cold period: October–March. Horizontal error bars represent the 95% CIs; where the CI would not fit on the figure an arrowhead is used to denote that it continues. Black dots represent ORs, grey squares represent weights.

Fig 2 summarizes the ORs for ED visits for drug and alcohol abuse related to exposure to the six air pollutants examined with lags of 0 (day of visit) to 7 days. The conditional logistic regression in all the constructed models was adjusted for ambient temperature and relative humidity. Both CO and NO_2_ displayed positive and statistically significant associations with ED visits. To investigate the possibility of seasonal variation in the effects of pollutants, the relationship between pollutants and ED visits for drug and alcohol abuse was examined in the cold (October-March, Fig 2, right-hand panel) and warm (April-September) periods. Effects of CO and NO_2_ were enhanced in the cold period, and an effect of PM was also observed for lags of 2 and 3 days for both PM_10_ and PM_2.5_. We observed no effects or even negative effects for ozone in both the overall analysis and in the analysis that considered the cold period alone. We did not observe significant associations for the warm period (April–September) but for two months (May–June) the results were positive and statistically significant: OR = 1.20 (1.07, 1.36) for CO and OR = 1.10 (1.00, 1.22) for NO_2_ both with lag 1. In the case of exposure to ground-level ozone in the warm period, the results were positive but not statistically significant. As shown in Table 4 ozone levels in the warm period are much higher comparing to levels in the cold period.

**Fig 3:**
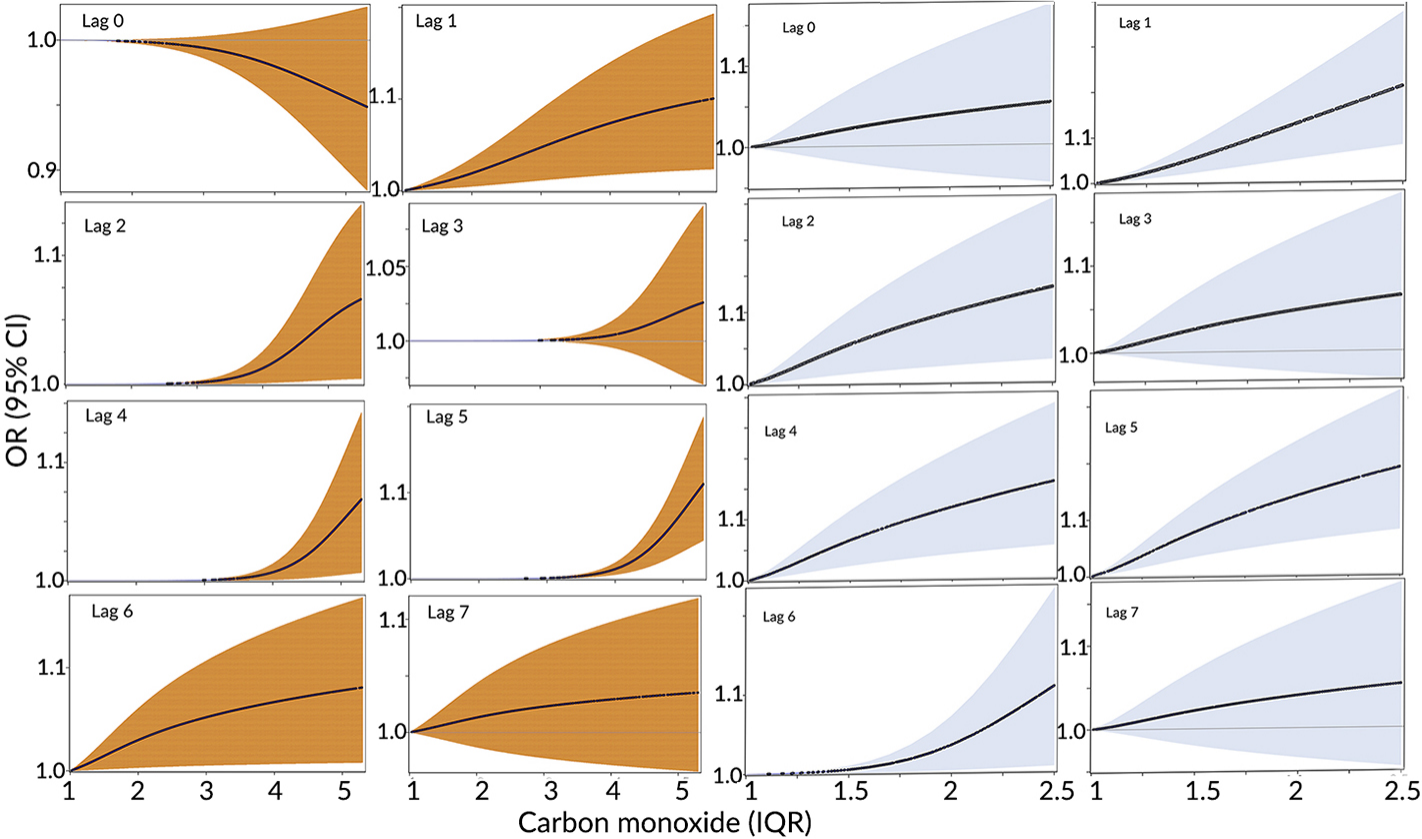
ORs and 95% CIs for ED visits for substance use disorders along IQR values for carbon monoxide. The ORs are reported for same day (0) as well as lags 1–7 days. Left-hand panel—whole period: January—December, right-hand panel—cold period: October–March. Colored areas represent the 95% CIs.

Fig 3 illustrates the results from non-linear parametric models for exposure to carbon monoxide. Positive associations persisted, and for the cold period the exposure-response relationship is very close to linear across lags. Overall, the results generated by this method agree with the results from the CC method on the statistical significance of associations.

**Fig 4:**
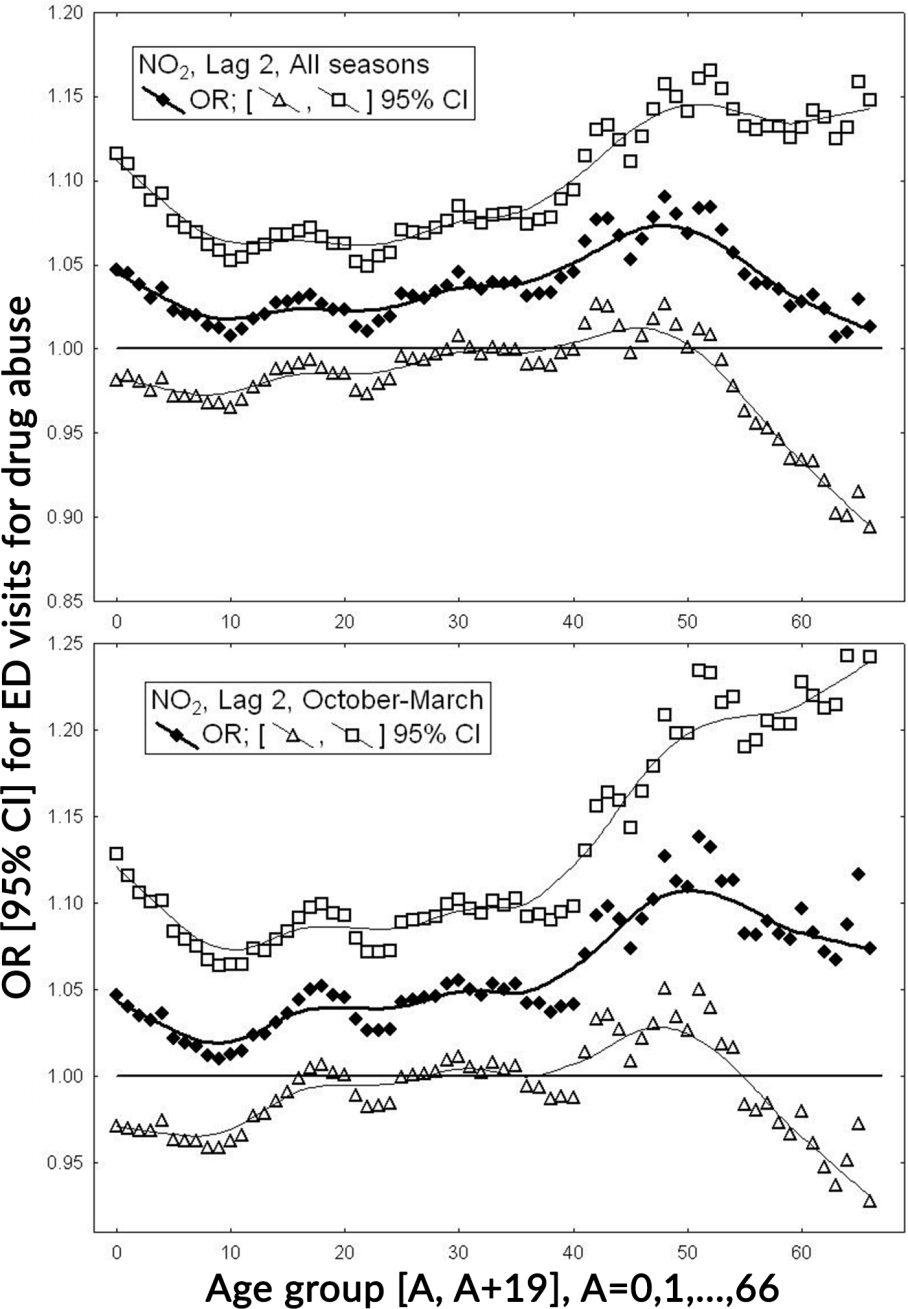
ORs and 95% CIs for ED visits for substance use disorders for one IQR value for nitrogen dioxide (NO_2_) lagged by 2 days. ORs are reported for 67 age groups. Upper panel—whole period (January–December), lower panel—cold period (October–March).

Fig 4 shows the estimated ORs and their 95% CIs for exposure to nitrogen dioxide lagged by 2 days. The figure shows odds ratios estimated separately for a sequence of 67 age groups. These age groups are defined as follows: [A, A+19], for A = 0, 1,…, 66. Upper panel of the figure represents the values for all seasons and lower panel for the cold period. We can compare among the defined groups and Fig 1 provides the number of the patients. The strongest association is observed for the cold period. The interaction term for the air pollution and season (warm = 0, cold = 1) was 1.05 (95%CI: 0.99, 1.11). The interaction term for sex (female = 0, male = 1) and exposure was estimated as 0.96 (95%CI: 0.95, 1.05) for whole period, and 1.02 (95%CI: 0.96, 1.08) for cold period.

## Discussion

This study examined visits for drug and alcohol abuse for patients in five EDs in Edmonton, Canada, over a period of 10 years. The principal result of this study is the estimation of an association between exposure to specific ambient air pollutants and the numbers of ED visits for substance use issues. These visits may represent acute psychiatric symptomology manifested as drug and/or alcohol abuse.

Carbon monoxide, nitrogen dioxide, and particulate matter were each individually associated with increased presentations for substance abuse. While there is currently no literature on the impact of air pollution on substance abuse, there is a growing body of evidence that exposure to air pollution affects the brain and can alter behaviour [12]. Several studies have reported associations between air pollutants and cognitive function [20–26]. Chronic exposure to traffic-related air pollution (NO_2_ and PM_10_ used as indicators) has been associated with decreased neurobehavioural function in children, and enrollment in learning disability programs was found to be higher in areas with elevated lead and air pollution levels [27]. Recent work has also shown associations between air pollution and criminal activity. Supporting these associations, mice exposed chronically to air pollution exhibit cognitive deficits and signs of depression [7]. Interestingly, a recent study demonstrated that controlled exposure of mice to concentrated ambient particles increased their preference for immediate reward [8], a trait associated with addiction-related behaviours [28]. Air pollutants rapidly activate the stress axis and increase glucocorticoid release in rats [10], with dysregulation of this system implicated in anxiety and depression [29–31]. Given the established link between depressive states and substance use and abuse [32], it seems plausible that the association of air pollution with hospital presentations for substance abuse may be a consequence of impacts of pollutants on mental health.

We observed stronger associations in the cold season than in the warm season. Seasonal variation in depression is believed to be affected by light, with people in higher latitudes more vulnerable to this factor as differences in light exposure by seasons are larger. One group [33] used the frequencies of internet key-word searches related to depression to show that people in higher latitudes were more impacted by seasonal change compared to those in tropical areas. Associations between air pollution and suicidal behaviour have been shown to be increased during the winter [4,34]; it may be that individuals prone to anxiety and/or depression self-medicate with drugs or alcohol during this time, resulting in increased rates of ED presentations.

In contrast with the positive association of CO, NO_2_ and particulate matter on presentations for substance abuse, ozone levels were inversely correlated. The association of ozone with decreased presentations for substance abuse may be attributable to effects of covariates. Generation of ground-level ozone occurs through photochemical reaction with oxides of nitrogen, carbon monoxide, and volatile organic compounds, and so there is a relationship between ozone levels and sunlight. As light therapy is associated with decreased depressive symptoms [35], it is possible that the inverse relationship between ozone levels and ED visits for substance abuse is due to the effect of sunlight rather than some beneficial effect of ozone inhalation.

As was already mentioned, there is growing evidence that air pollution can contribute to cognitive and psychiatric disorders. Exposure to ambient air pollution may trigger various health conditions and behaviours [36,37], which may depend upon interindividual differences in susceptibility. The growing list of health problems thought to be exacerbated by exposure to air pollution include psychiatric conditions [38] and suicide [39]. Additional work is warranted to investigate causal links in the pathway from air pollution exposure to these adverse health outcomes.

There are various limitations that are typical of this type of research, including the adequacy of the models used and the impact of measurement error in the exposure and outcome variables. The largest distance among the three monitors is about 11 km. The five hospitals participated in the study are not separated from each other by more than 20 km. The error in estimating personal exposure from three fixed-site monitor stations would tend to reduce the probability of detecting an effect and, in most cases, bias air pollution–ED visits for substance abuse correlations toward the null [40]. Another important limitation is the simplified assumption that each person has the same exposure; exposure may vary according to location of home and workplace, occupational setting, time spent outdoors, and other factors. PM_2.5_ data were available for a limited time period, which reduces power to detect associations with this pollutant. Misclassifications of the cause of ED visits or underreporting in the hospital registry system might also have confounded the results. The measured values for carbon monoxide may not represent real vertical distribution of levels. Carbon monoxide is slightly less dense than air, with a molar mass of 28.0 vs 28.8 average molar mass of air. Consequently, ground level exposure concentrations may be higher than those collected by monitoring stations as these are usually located a few meters above the ground.

## Conclusions

There is a growing toxicological literature showing that exposure to gaseous and particulate air pollutants can cause adverse neurological effects ranging from behavioural changes to neurodegeneration. This evidence, mainly from experimental animal studies, provides biological plausibility for the hypothesis that exposure of human populations to air pollutants, in concert with susceptibility factors related to age, disease or genetics, may result in neurochemical or neuropathological changes. Such effects could potentially manifest as or contribute to depression or related psychological outcomes [9,41–51], which could result in increased substance abuse. The results of the present study suggest that variation in levels of ambient air pollutants may be associated with the number of ED visits for substance abuse.

## References

[1] Lundberg A. Psychiatric aspects of air pollution. Otolaryngol Head Neck Surg. 1996;114, 227–231. doi: 10.1016/S0194-59989670172-9 8637739 10.1016/S0194-59989670172-9

[2] Kim C, Jung SH, Kang DR, Kim HC, Moon KT, Hur NW, et al Ambient particulate matter as a risk factor for suicide. Am J Psychiatry. 2010;167(9), 1100–1107. doi: 10.1176/appi.ajp.2010.09050706 20634364 10.1176/appi.ajp.2010.09050706

[3] Szyszkowicz M, Kousha T, Kingsbury M, Colman I. Air Pollution and Emergency Department Visits for Depression: A Multicity Case-Crossover Study. Environmental Health Insights. 2016;10, 155–161. doi: 10.4137/EHI.S40493 27597809 10.4137/EHI.S40493 PMC5006648

[4] Szyszkowicz M, Willey JB, Grafstein E, Rowe BH, Colman I. Air pollution and emergency department visits for suicide attempts in Vancouver, Canada. Environ Health Insights.2010; 4, 79–86. doi: 10.4137/EHI.S5662 21079694 10.4137/EHI.S5662 PMC2978939

[5] Szyszkowicz M, Kaplan GG, Grafstein E, Rowe BH. Emergency department visits for migraine and headache: a multi-city study. Int J Occup Med Environ Health. 2009;22(3), 235–242. doi: 10.2478/v10001-009-0024-5 19819836 10.2478/v10001-009-0024-5

[6] Biermann T, Stilianakis N, Bleich S, Thürauf N, Kornhuber J, Reulbach U. The hypothesis of an impact of ozone on the occurrence of completed and attempted suicides. Med Hypotheses. 2009;72(3), 338–341. doi: 10.1016/j.mehy.2008.09.042 19027246 10.1016/j.mehy.2008.09.042

[7] Fonken LK, Xu X, Weil ZM, Chen G, Sun Q, Rajagopalan S, et al Air pollution impairs cognition, provokes depressive-like behaviors and alters hippocampal cytokine expression and morphology. Mol Psychiatry. 2011;16(10), 987–995. doi: 10.1038/mp.2011.76 21727897 10.1038/mp.2011.76 PMC3270364

[8] Allen JL, Conrad K, Oberdörster G, Johnston CJ, Sleezer B, Cory-Slechta DA. Developmental Exposure to Concentrated Ambient Particles and Preference for Immediate Reward in Mice. Environ Health Perspect. 2013;121, 32–38. http://dx.doi.org/10.1289/ehp.1205505 [Online 11 October 2012]. 23063827 10.1289/ehp.1205505 PMC3553438

[9] Thomson EM. Neurobehavioral and metabolic impacts of inhaled pollutants. A role for the hypothalamic-pituitary-adrenal axis? Endocrine Disruptors. 2014;1(1). doi: 10.4161/endo.25066

[10] Thomson EM, Vladisavljevic D, Mohottalage S, Kumarathasan P, Vincent R. Mapping acute systemic effects of inhaled particulate matter and ozone: multiorgan gene expression and glucocorticoid activity. Toxicol. Sci. 2013;135(1), 169–181. doi: 10.1093/toxsci/kft137 23805001 10.1093/toxsci/kft137 PMC3748763

[11] Thomson EM, Pal S, Guénette J, Wade MG, Atlas E, Holloway AC, et al Ozone Inhalation Provokes Glucocorticoid-Dependent and -Independent Effects on Inflammatory and Metabolic Pathways. Toxicol Sci. 2016;152(1), 17–28. doi: 10.1093/toxsci/kfw061 27037194 10.1093/toxsci/kfw061 PMC12077420

[12] Block ML, Elder A, Auten RL, Bilbo SD, Chen H, Chen JC, et al The outdoor air pollution and brain health workshop. Neurotoxicology. 2012;33(5), 972–84. doi: 10.1016/j.neuro.2012.08.014 Epub 2012 Sep 5. 22981845 10.1016/j.neuro.2012.08.014 PMC3726250

[13] Matsushita S, Higuchi S. Alcohol-related disorders and suicide. Seishin Shinkeigaku Zasshi. 2009;111(10), 1191–1202. 20058674

[14] Lukassen J, Beaudet MP. Alcohol dependence and depression among heavy drinkers in Canada. Soc Sci Med. 2005;61(8), 1658–1667. doi: 10.1016/j.socscimed.2005.03.019 15869834 10.1016/j.socscimed.2005.03.019

[15] Amitai Y, Zlotogorski Z, Golan-Katzaw V, Wexler A, Gross D. Neuropsychological impairment from acute low-level exposure to carbon monoxide. Arch Neurol. 1998;55, 845–848. 9626776 10.1001/archneur.55.6.845

[16] Maclure M. The case-crossover design. A method for studying transient effects on the risk of acute events. Am J Epidemiol. 1991;133, 144–153. 1985444 10.1093/oxfordjournals.aje.a115853

[17] Nasari MM, Szyszkowicz M, Chen H, Crouse D, Turner MC, Jerrett M, et. al A class of non-linear exposure-response models suitable for health impact assessment applicable to large cohort studies of ambient air pollution. Air Quality, Atmosphere & Health. 2016; 9(8), 961–972. 10.1007/s11869-016-0398-z PMC5093184 27867428

[18] Wang SV, Coull BA, Schwartz J, Mittleman MA, Wellenius GA. Potential for bias in case-crossover studies with shared exposures analyzed using SAS. Am J Epidemiol. 2011;174, 118–124 doi: 10.1093/aje/kwr038 21540322 10.1093/aje/kwr038 PMC3133813

[19] Janes H, Sheppard L, Lumley T. Case-crossover analyses of air pollution exposure data. Referent selection strategies and their implications for bias. Epidemiology. 2005;16, 717–726. 16222160 10.1097/01.ede.0000181315.18836.9d

[20] Calderón-Garcidueñas L, Mora-Tiscareño A, Ontiveros E, Gómez-Garza G, Barragán-Mejía G, Broadway J, et al Air pollution, cognitive deficits and brain abnormalities: a pilot study with children and dogs. Brain Cogn.2008;68(2), 117–127. doi: 10.1016/j.bandc.2008.04.008 18550243 10.1016/j.bandc.2008.04.008

[21] Chen JC, Schwartz J. Neurobehavioral effects of ambient air pollution on cognitive performance in US adults. Neurotoxicology. 2009;30, 231–239. doi: 10.1016/j.neuro.2008.12.011 19150462 10.1016/j.neuro.2008.12.011

[22] Power MC, Weisskopf MG, Alexeeff SE, Coull BA, Spiro A 3rd, Schwartz J. Traffic-related air pollution and cognitive function in a cohort of older men. Environ. Health Perspect. 2011;119, 682–687. doi: 10.1289/ehp.1002767 21172758 10.1289/ehp.1002767 PMC3094421

[23] Ranft U, Schikowski T, Sugiri D, Krutmann J, Krämer U. Long-term exposure to traffic-related particulate matter impairs cognitive function in the elderly. Environ. Res. 2009;109, 1004–1011. doi: 10.1016/j.envres.2009.08.003 19733348 10.1016/j.envres.2009.08.003

[24] Suglia SF, Gryparis A, Wright RO, Schwartz J, Wright RJ. Association of black carbon with cognition among children in a prospective birth cohort study. Am. J. Epidemiol. 2008;167, 280–286. doi: 10.1093/aje/kwm308 18006900 10.1093/aje/kwm308

[25] Wang S, Zhang J, Zeng X, Zeng Y, Wang S, Chen S. Association of traffic-related air pollution with children's neurobehavioral functions in Quanzhou, China. Environ. Health Perspect. 2009;117, 1612–1618. doi: 10.1289/ehp.0800023 20019914 10.1289/ehp.0800023 PMC2790518

[26] Weuve J, Puett RC, Schwartz J, Yanosky JD, Laden F, Grodstein F. Exposure to particulate air pollution and cognitive decline in older women. Arch Intern Med. 2012;172(3), 219–227. doi: 10.1001/archinternmed.2011.683 22332151 10.1001/archinternmed.2011.683 PMC3622279

[27] Margai F, Henry N. A community-based assessment of learning disabilities using environmental and contextual risk factors. Soc Sci Med. 2003;56(5), 1073–1085. 12593879 10.1016/s0277-9536(02)00104-1

[28] Bickel WK, Jarmolowicz DP, Mueller ET, Koffarnus MN, Gatchalian KM. Excessive discounting of delayed reinforcers as a trans-disease process contributing to addiction and other disease-related vulnerabilities: emerging evidence. Pharmacol Ther.2012;134(3), 287–297. doi: 10.1016/j.pharmthera.2012.02.004 22387232 10.1016/j.pharmthera.2012.02.004 PMC3329584

[29] Pruessner M, Hellhammer DH, Pruessner JC, Lupien SJ. Self-reported depressive symptoms and stress levels in healthy young men: associations with the cortisol response to awakening. Psychosomatic Med. 2003;65(1), 92–99. 10.1097/01.psy.0000040950.22044.10 12554820

[30] Mitra R, Sapolsky RM. Acute corticosterone treatment is sufficient to induce anxiety and amygdaloid dendritic hypertrophy. Proc. Natl. Acad. Sci. 2008;105(14), 5573–5578. doi: 10.1073/pnas.0705615105 18391224 10.1073/pnas.0705615105 PMC2291109

[31] Lenze EJ, Mantella RC, Shi P, Goate AM, Nowotny P, Butters MA, et al Elevated cortisol in older adults with generalized anxiety disorder is reduced by treatment: a placebo-controlled evaluation of escitalopram. Am J Geriatr Psychiatry. 201;19(5):482–90. doi: 10.1097/JGP.0b013e3181ec806c 20808146 10.1097/JGP.0b013e3181ec806c PMC3424606

[32] Fergusson DM, Boden JM, Horwood LJ. Tests of causal links between alcohol abuse or dependence and major depression. Arch. Gen. Psychiatry.2009;66(3), 260–266. doi: 10.1001/archgenpsychiatry.2008.543 19255375 10.1001/archgenpsychiatry.2008.543

[33] Yang AC, Huang NE, Peng C-K, Tsai S-J. Do Seasons Have an Influence on the Incidence of Depression? The Use of an Internet Search Engine Query Data as a Proxy of Human Affect. PLoS ONE. 2010; 5(10): e13728 doi: 10.1371/journal.pone.0013728 21060851 10.1371/journal.pone.0013728 PMC2965678

[34] Makris GD, Reutfors J, Osby U, Isacsson G, Frangakis C, Ekbom A, Papadopoulos FC. Suicide seasonality and antidepressants: a register-based study in Sweden. Acta Psychiatr Scand. 2012;127(2), 117–125. doi: 10.1111/j.1600-0447.2012.01891.x 22676408 10.1111/j.1600-0447.2012.01891.x

[35] Partonen T, Lönnqvist J. Prevention of winter seasonal affective disorder by bright-light treatment. Psychol Med. 1996;26(5), 1075–1080. 8878339 10.1017/s003329170003539x

[36] Sussman S, Ames SL, Evol E. Could environmental exposures facilitate the incidence of addictive behaviors. Eval Health Prof. 2015;38(1)53–58. doi: 10.1177/0163278713501692 23970431 10.1177/0163278713501692 PMC4045655

[37] Lu JG, Lee JJ, Gino F, Galinsky AD. Polluted Morality: Air Pollution Predicts Criminal Activity and Unethical Behavior. Psychol Sci. 2018;29(3):340–355. doi: 10.1177/0956797617735807 29412050 10.1177/0956797617735807

[38] Casas L, Cox B, Bauwelinck M, Nemery B, Deboosere P, Nawrot TS. Does air pollution trigger suicide? A case-crossover analysis of suicide deaths over the life span. Eur J Epidemiol. 2017 11;32(11):973–981. doi: 10.1007/s10654-017-0273-8 28623424 10.1007/s10654-017-0273-8

[39] Oudin A, Åström DO, Asplund P, Steingrimsson S, Szabo Z, Carlsen HK. The association between daily concentrations of air pollution and visits to a psychiatric emergency unit: a case-crossover study. Environ Health. 2018 1 10;17(1):4 doi: 10.1186/s12940-017-0348-8 29321054 10.1186/s12940-017-0348-8 PMC5763570

[40] Zeger SL, Thomas D, Dominici F, Samet JM, Schwartz J, Dockery D., et al Exposure measurement error in time-series studies of air pollution: concepts and consequences. Environ Health Perspect. 2000;108,419–426. 10811568 10.1289/ehp.00108419 PMC1638034

[41] Szyszkowicz M, Rowe B, Colman I. Air pollution and daily emergency department visits for depression. Int J Occup Med Environ Health. 2009;22(4), 355–362. doi: 10.2478/v10001-009-0031-6 20197262 10.2478/v10001-009-0031-6

[42] Dales RE, Cakmak S, Vidal CB. Air pollution and hospitalization for headache in Chile. Am J Epidemiol. 2009;70, 1057–1066 10.1093/aje/kwp217 PMC2755178 19741041

[43] Lee WJ, Alavanja MCR, Hoppin JA, Rusiecki JA, Kamel F, Blair A, et al Mortality among pesticide applicators exposed to chlorpyrifos in the Agricultural Health Study. Environ Health Perspect. 2007;115, 528–534. doi: 10.1289/ehp.9662 17450220 10.1289/ehp.9662 PMC1852666

[44] Stallones L. Suicide and potential occupational exposure to pesticides, Colorado 1990–1999. Journal-of-Agromedicine. 2006;11, 107–112. doi: 10.1300/J096v11n03_11 19274902 10.1300/J096v11n03_11

[45] Bakian AV, Huber RS, Coon H, Gray D, Wilson Ph, McMahon WM, et al Acute Air Pollution Exposure and Risk of Suicide Completion. Am J Epidemiol. 2015;181(5). 295–303. doi: 10.1093/aje/kwu341 25673816 10.1093/aje/kwu341 PMC4339389

[46] Herrnstadt E, Muehlegger E. Air Pollution and Criminal Activity: Evidence from Chicago Microdata. National Bureau of Economic Research. 2015. NBER Working Paper No. 21787. doi: 10.3386/w21787

[47] Petridou E, Papadopoulos FC, Frangakis CE, Skalkidou A, Trichopoulos D. A role of sunshine in the triggering of suicide. Epidemiology. 2002;13, 106–109. 11805594 10.1097/00001648-200201000-00017

[48] van Wijngaarden E. An exploratory investigation of suicide and occupational exposure. J Occup Environ Med. 2003;45, 96–101. 12553184 10.1097/00043764-200301000-00018

[49] Łopuszańska U, Makara-Studzińska M. The correlations between air pollution and depression, Current Problems of Psychiatry. 2017;18(2)2,100–109.

[50] Szyszkowicz M, Rowe BH. Ambient air pollution and depressive symptoms. PeerJ PrePrints. 2014 https://doi.org/10.7287/peerj.preprints.757v1.

[51] Chen JC, Samet JM. Air pollution and suicide risk: another adverse effect of air pollution? Eur J Epidemiol. 2017 11;32(11):943–946. doi: 10.1007/s10654-017-0329-9 29101595 10.1007/s10654-017-0329-9 PMC5786371

